# Carbon plates in running shoes biomechanics: a systematic review and meta-analysis

**DOI:** 10.3389/fspor.2026.1764338

**Published:** 2026-05-29

**Authors:** Sofia Giachetti Martin, Eiki Nicholas Kobayashi, Laura Amaral Coelho de Azevedo, David Bonini Vieira Campanhã, Diego Escudeiro de Oliveira, Pedro Baches Jorge

**Affiliations:** 1 Faculdade de Ciências Médicas da Santa Casa de São Paulo, Medical School, São Paulo, Brazil; 2 Department of Orthopedics and Traumatology, Irmandade da Santa Casa de Misericórdia de São Paulo, São Paulo, Brazil

**Keywords:** ankle power, biomechanics, carbon plated shoes, longitudinal bending stiffness, running

## Abstract

**Background:**

Carbon-plated running shoes (CPS) combine compliant foams with embedded carbon-fiber plates to increase longitudinal bending stiffness and potentially improve running economy. However, whether CPS systematically alter running biomechanics compared with non-carbon-plated shoes (NCPS) remains unclear.

**Objective:**

To quantify the biomechanical effects of CPS in healthy adults.

**Methods:**

A systematic review and meta-analysis of crossover trials comparing CPS vs. NCPS was conducted according to PRISMA guidelines (PROSPERO CRD420251058609). Searches were performed in MEDLINE, Scopus, LILACS, and Embase (July 2025). Eligible studies included healthy adults (18–70 years) running in CPS and NCPS. Outcomes were step frequency (steps·min^−^¹), leg stiffness (kN·m^−^¹), and peak positive joint power (W·kg^−^¹) at the metatarsophalangeal (MTP), ankle, knee, and hip. Random-effects models were used to estimate standardized mean differences (SMDs) with 95% confidence intervals (CIs). Certainty of evidence was assessed using GRADE.

**Results:**

Fifteen studies were included. No significant differences were observed between CPS and NCPS for leg stiffness (SMD = −0.12; 95% CI: −0.46 to 0.23; *p* = 0.44), knee power (SMD = 0.21; 95% CI: −0.10 to 0.52; *p* = 0.12), hip power (SMD = −0.23; 95% CI: −1.36 to 0.90; *p* = 0.56), or MTP power (SMD = 0.13; 95% CI: −1.87 to 2.12; *p* = 0.85). A borderline reduction in ankle power was observed with CPS (SMD = −0.71; 95% CI: −1.42 to 0.00; *p* = 0.05). Step frequency showed a small, non-significant decrease (SMD = −0.16; 95% CI: −0.32 to 0.01; *p* = 0.06). Certainty ranged from low to moderate.

**Conclusion:**

CPS do not produce consistent changes in joint power or leg stiffness compared with NCPS. Biomechanical adaptations appear subtle, with possible distal joint effects but no systematic redistribution of lower-limb mechanics.

**Systematic review registration:**

PROSPERO CRD420251058609.

## Introduction

With the advancement of sports science, running has become a central area for the development of technologies aimed at improving biomechanical efficiency and endurance performance. In recent years, manufacturers have introduced models incorporating highly compliant foams and embedded carbon-fiber plates within the midsole. One of the earliest widely adopted examples was released in 2017 and featured a combination of lightweight foam and a stiff plate designed to increase longitudinal bending stiffness. This configuration was proposed to improve running economy by enhancing energy return, facilitating forward propulsion, and reducing muscular demand during long-distance running (1). Notably, several elite athletes tested this shoe model and subsequently set new world records in 100 km, marathon (42 km), half-marathon (21 km), and 15 km events.

In running biomechanics, several parameters are analyzed, including step frequency (steps·min^−^¹), stride length (m), power output (W·kg^−^¹), mechanical work (J·kg^−^¹), angular velocity (°·s^−^¹), joint angles (°), joint moments at the hip, knee, ankle, and metatarsophalangeal joints, as well as ground contact time (s), flight time (s), and leg stiffness (kN·m^−^¹) (2).

Evidence from recent studies indicates that carbon-plated running shoes, when compared to non-carbon models, can reduce the mechanical workload at the ankle joint (3), redistribute joint demands toward the knee and hip (4), and consequently attenuate fatigue in the distal lower limb, such as the ankle. Moreover, the carbon plate appears to decrease energy dissipation, which has been associated with improvements in running economy and propulsive efficiency in previous experimental studies (1, 5, 25, 30, 32, 35). Additionally, some studies have reported alterations in step frequency and stride length, typically characterized by longer strides and lower cadence, which may be advantageous depending on individual running mechanics (1, 6).

With the increasing adoption of shoe designs that combine compliant foams and embedded carbon-fiber plates, interest has grown in understanding whether these features meaningfully alter running biomechanics. Previous studies have proposed that increased longitudinal bending stiffness and optimized midsole geometry may influence joint power, leg stiffness, and spatiotemporal parameters. However, findings across the literature remain inconsistent (27, 32). Therefore, the objective of this systematic review and meta-analysis was to evaluate whether carbon plated shoes (CPS) produce measurable changes in selected biomechanical outcomes compared with non-carbon plated shoes (NCPS). Specifically, the review assessed joint power at the hip, knee, ankle, and metatarsophalangeal joints, leg stiffness, and key spatiotemporal variables such as stride frequency.

Carbon-plated running shoes have been consistently associated with improvements in running economy and reductions in metabolic cost, as demonstrated in a recent systematic review with meta-analysis focusing on energetic outcomes. Kobayashi et al. reported robust and consistent metabolic benefits of carbon-plated footwear across different running speeds and populations (7). However, metabolic and physiological outcomes do not necessarily mirror biomechanical adaptations, and improvements in running economy may arise from mechanisms not fully captured by joint-level mechanical variables. For this reason, the present review was deliberately designed to focus exclusively on biomechanical outcomes, including joint power, leg stiffness, and spatiotemporal parameters. Energetic and physiological variables (e.g., VO₂, metabolic cost, mechanical efficiency) were not included, as their integration would require distinct methodological approaches and outcome hierarchies, potentially obscuring the specific biomechanical question addressed herein. Consequently, this review does not aim to explain performance or metabolic mechanisms, but rather to determine whether consistent biomechanical alterations accompany the use of carbon-plated running shoes.

## Methods

### Protocol and registration

A systematic review with meta-analysis was conducted in accordance with the Preferred Reporting Items for Systematic Reviews and Meta-Analyses (PRISMA) 2020 guidelines. The review protocol was prospectively registered and approved in the International Prospective Register of Systematic Reviews (PROSPERO; CRD420251058609).

### Identification and selection of the studies

Two independent reviewers conducted a structured search in PubMed/MEDLINE, Embase, Scopus, and the Virtual Health Library (BVS), using combinations of DeCS/MeSH descriptors related to “shoes,” “running,” and “biomechanics”. Table 1 presents the complete search strategy for each database. Full-text articles meeting the inclusion criteria were thoroughly assessed, and data were extracted into a standardized spreadsheet (Microsoft Excel™). An independent reviewer (S.G.M.) independently screened titles and abstracts. A second independent reviewer (E.N.K.) the evaluated and judges the selection of studies and disagreements were resolved by consensus. Then, full texts of the selected articles were collected and evaluated at the same manner.

**Table 1:** Search strategies.

Searching terms	Running AND Shoes AND Biomechanics	Running AND Shoes AND Biomechanics	Running AND Shoes AND Biomechanics	Running AND Shoes AND Biomechanics
Articles found	889	1250	933	6
Seach strategy	(“running"[MeSH Terms] OR “running"[All Fields] OR “runnings"[All Fields]) AND (“shoe s"[All Fields] OR “shoeing"[All Fields] OR “shoes"[MeSH Terms] OR “shoes"[All Fields]) AND (“biomechanical phenomena"[MeSH Terms] OR (“biomechanical"[All Fields] AND “phenomena"[All Fields]) OR “biomechanical phenomena"[All Fields] OR “biomechanic"[All Fields] OR “biomechanics"[All Fields] OR “biomechanical"[All Fields] OR “biomechanically"[All Fields])	TITLE-ABS-KEY (running AND shoes AND biomechanics)	running shoes biomechanics’ OR ((‘running'/exp OR running) AND (‘shoes'/exp OR shoes) AND (‘biomechanics'/exp OR biomechanics))	running AND shoes AND biomechanics
Data base	PubMed	Scopus	Embase	Lilacs
Total (n)	44	36	23	0
N° selected studies for full review	44	36	23	0

Search strategies used for the literature search across four databases (PubMed, Scopus, Embase, Lilacs). Describes search terms, filters, and total records retrieved from each source.

Language was restricted to English, with no restrictions on publication date. Inclusion criteria were:

**-** Study Type: Comparative studies randomized or non-randomized, that evaluated running with carbon-plated shoes vs. non-carbon-plated shoes.
**-** Population: Healthy adults (18-70 years), recreational or professional athletes.
**-** Intervention: Running shoes with carbon plates.
**-** Comparators: Running shoes without carbon plates.
**-** Outcomes (meta-analysis parameters): Step frequency (steps/min), Leg stiffness (kN/m), Peak positive power (W/kg) at the metatarsophalangeal (MTP), knee, ankle, and hip joints

Studies were excluded when biomechanical outcomes were not reported in absolute units or formats compatible with quantitative synthesis. Specifically, studies reporting leg stiffness using normalized or dimensionless spring-mass model outputs, spatiotemporal variables presented only as relative changes or interaction effects, or lacking extractable measures of central tendency and variability (e.g., means with standard deviations, standard errors, or confidence intervals) for the predefined biomechanical endpoints were not eligible for meta-analysis. The study was included if it contained at least one of the listed outcomes. After meeting the inclusion criteria, the final articles were selected for data extraction.

### Data extraction

One investigator (S.G.M.) independently extracted the main data from the selected articles screened titles and abstracts. Data was extracted into a standardized spreadsheet (Microsoft Excel™). Extracted data included study characteristics, participant demographics, sample size, shoe model specifications (mass, stiffness, midsole material, presence/absence of carbon plate), running speed, testing environment, and main biomechanical parameters analyzed in each study. Authors of the included articles were contacted to request missing information when necessary.

### Risk of bias assessment

Two independent reviewers (S.G.M. and E.N.K) assessed the risk of bias for all included articles. The Risk of Bias in Systematic Reviews (ROBIS) tool was employed. Phase 2 domains (study selection, identification, data collection, quality assessment) and Phase 3 domains (synthesis and findings) were rated as low, high, or unclear risk.

### Certainty of evidence

The GRADE (Grading of Recommendations, Assessment, Development, and Evaluation) approach, adapted for overview studies, was used to assess risk of bias, inconsistency, imprecision, indirectness, and publication bias. Two independent reviewers (S.G.M. and E.N.K) conducted the assessment, with disagreements resolved by consensus or consultation with a third reviewer.

### Data analysis

Meta-analyses were performed using RevMan Web (version 4.0, Cochrane Collaboration) in combination with the meta (v6.5-0) and metafor (v4.5-0) packages. The Standardized Mean Difference (SMD) with 95% confidence intervals (CIs) was calculated for each outcome. A random-effects model was adopted due to the expected heterogeneity among included studies. Between-study variance (*τ*²) was estimated using the Restricted Maximum-Likelihood (REML) method, and CI uncertainty was adjusted with the Hartung–Knapp–Sidik–Jonkman (HKSJ) approach. Heterogeneity was evaluated using Cochran's Q (*χ*²) test and the I² statistic, which quantifies the proportion of total variability due to between-study heterogeneity.

Separate forest plots were generated for each outcome of interest, which resulted in 5 meta-analyses. The level of statistical significance was set at *p* < 0.05. To facilitate comprehension and observation of the biomechanics variations provided by the carbon plate shoe condition, the results were synthesized with the average percentage of variation between the conditions: carbon plate shoe vs. non-carbon plate shoe.

## Results

### Flow of studies

A total of 3078 records were identified across the four databases (PubMed, Scopus, Lilacs, and Embase). After removing 38 duplicates, 3,040 records were screened by title and abstract, of which 2,977 were excluded. Sixty-three full-text articles were assessed for eligibility, and 47 were excluded for the following reasons: not comparing shoes with and without a carbon plate (*n* = 9), not using carbon-plated shoes (*n* = 13), not analyzing running biomechanics (*n* = 2), non-comparative study design (*n* = 3), comparing carbon plates in different shoe locations (*n* = 1), full text available only in Dutch (*n* = 1), not using conventional running footwear (*n* = 1), absence of a results table (*n* = 2), and not analyzing the pre-specified biomechanical outcomes (*n* = 15).

One potentially eligible study, Durante et al. (8), was not publicly accessible. The corresponding authors were contacted, however, no response was received. Therefore, it was not included in the meta-analysis. Additionally, two other studies: Cigoja et al. (9) and Miyazaki et al. (5) were excluded, because the data regarding the outcomes could not be extracted. In the Cigoja et al. (9) study, the only information about the joint power, were curves across the stance phase, precluding its use for the meta-analysis. Regarding Miyazaki et al. (5), the MTP joint power data were insufficient, as no numerical values were displayed in a table. Only a graphical representation of joint and segment power during the stance phase of running was presented, compromising its use for the meta- analysis.

**Figure 1:**
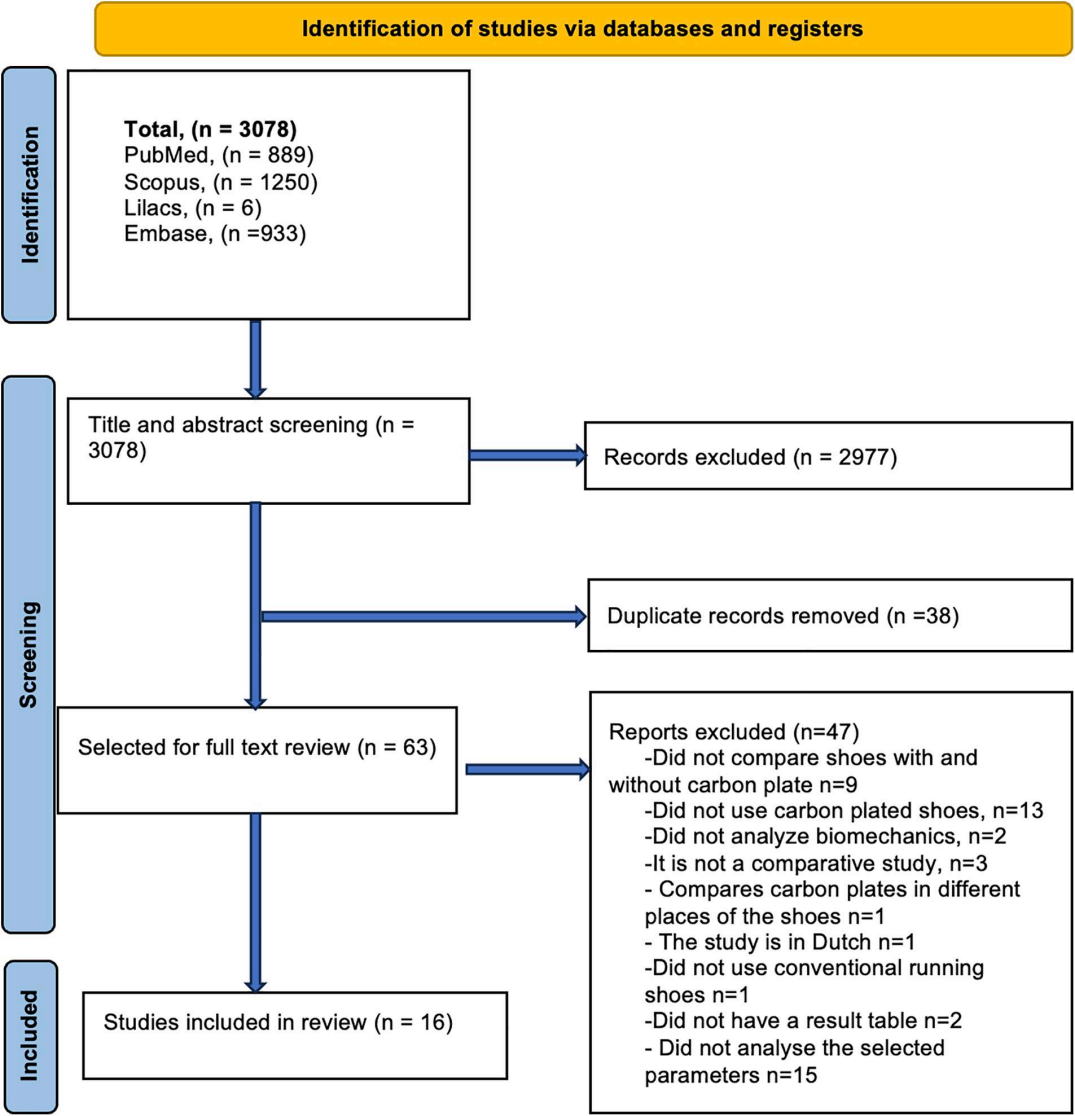
Prisma flow diagram. A total of 3,078 records were identified through database searching (PubMed *n* = 889; Scopus *n* = 1,250; Embase *n* = 933; LILACS *n* = 6). After removal of 38 duplicate records, titles and abstracts of 3,078 records were screened, and 2,977 were excluded. Sixty-three full-text articles were assessed for eligibility. Of these, 47 reports were excluded for the following reasons: no comparison between carbon-plated and non-carbon-plated shoes (*n* = 9); no use of carbon-plated footwear (*n* = 13); no biomechanical analysis (*n* = 2); non-comparative study design (*n* = 3); comparison of carbon plates in different shoe locations (*n* = 1); non-eligible language (Dutch) (*n* = 1); use of non-conventional footwear (*n* = 1); absence of results table (*n* = 2); and failure to analyze the predefined biomechanical parameters (*n* = 15). A total of 16 studies were included in the final review.

A total of 16 studies were therefore included in the final review. See complete flow of studies in Figure 1.

### Study characteristics

Characteristics of the included studies are showed in Table 2. Experimental studies with a randomized crossover design, published between 2018 and 2024, were included. To clarify, no articles published in 2025 were included in the meta-analysis, as they did not meet our search criteria. Sample sizes ranged from 9 to 28 runners, predominantly male, with ages spanning from young adults to experienced older athletes. The most frequently used control footwear included *Adidas Adizero Adios Boost 2*, *Nike Pegasus*, *Nike Infinity Run*, and standardized laboratory models, with mass between 199 and 240 g and stiffness ranging from 1.2 to 19.1 N/mm. The CPS comprised both experimental prototypes and commercial models, such as the *Nike ZoomX Vaporfly Next% 2*, with mass between 201 and 289 g and stiffness values ranging from 11.9 N/mm to 84.1 kN/m. Testing protocols were conducted either on a treadmill (8-12.6 km/h) or an athletics track (17.8-18.3 km/h), and assessed kinematic, kinetic, and spatiotemporal parameters, as well as leg stiffness and joint power.

**Table 2:** Characteristics of the studies included in the systematic review comparing biomechanical outcomes between carbon-plated and non-carbon-plated running shoes.

Study	Beltran et al	Castellanos-Salamanca et al. (1)	Castellanos-Salamanca et al. (2)	Flores et al	Hannigan et al	Hata et al. (1)	Hata et al. (2)	Hebert-Losier et al	Hoogkamer et al	Liu et al	Martinez et al	Perrin et al. (1)	Rodrigo-Carranza et al. (1)	Rodrigo-Carranza et al. (2)	Willwacher et al. (1)	Paradisis et al
Sample Size (n)	9 (4 women, 5 men)	12 male	12 men	19 male	12 (10 men, 2 women)	20 male	7 male	16 male	10 male	12 male	18 female	21 male	28 male	21 male	19 MALE	10 runners
Participants’ Characteristics	67 ± 3 with (26 years ± 13) of running experience	trained runner, 26.2 ± 8.1 years;	trained, 2.91 ± 7.50 years	recreational runners, 24 ± 6 years	at leats 3 times per week runners, 18–40 years	long distance runners, 20.7 ± 0.9 years	long distance runners, 20.1 [1.6] years	recreational runners, 3 ± 12.0 years	sub 35 min (10 km), 26.2 ± 4.0 years	recreational runners, 27:0 2:3 years	competitive runners, 18–45 years, ran 4 timeas a week	trained runners, 34 ± 9 yr;	runners, 28.1 ± 6.4 yr	runners at leats 3 times per week, 27.6 ± 5.2 years	SPORTING ACTIVITIES INCLUDING RUNNING ON A DAILY BASIS (25.3 ± 2.2 y)	recreational athletes, age (23.3 y (± 1.1)
Participants’ mass (kg)	69.5 ± 9.6	61.7 ± 4.3 kg	69.29 ± 7.55 kg	70.9 ± 4.1 kg,	not reported	57.2 ± 4.9 kg	57.1 [5.1] kg	77.0 ± 8.7 kg	63.2 ± 3.1 kg	67:5 4:5 kg	not reported	69.3 ± 4.6	64.8 ± 5.6 kg	65.3 ± 5.7 kg	74.9 ± 7.4 kg)	57.1 kg (± 3.8)
VO2max (mL·kg−1·min−1)	24.6 ± 3.5	not reported	not reported	control (vo2/min): 2.97 (0.36), HLBS (vo2/min0 2.98 (0.34)	not reported	not reported	not reported	not reported	not reported	with (40.92–3.68) without (42.06–3.65)	super (40.93 ± 3.15), control (42.59 ± 2.94)	60.3 ± 4.1	not reported	not reported	not reported	not reported
Non carbon plated shoe model	standard laboratory shoe (“Run Confort Noir,” Kalenji, Decathlon, France)	specifically manufactured for this study	Nike Pegasus 36 and 38, Nike Infinity Run 2, Nike Streak 6, Saucony Endorphin Speed 2, Joma R4000 and New Balance Hanzo S.	prototype shoes	New Balance Minimus 10v2	traditional shoe (MEDIFOAM Melos MF-003)	Nike Air Zoom Pegasus 34	Saucony Endorphin Racer 2	adidas adizero Adios BOOST 2	existing running shoe (Li-Ning Feidian, Li-Ning Company, Beijing, China	Nike Pegasus 38TM,	Kiprun KS900	experimental shoe model	experimental shoe model (diferrent from the carbon inserted shoes) but with similar features	Nike Free	Cohesion 13 shoe
Non carbon plated shoe mass(g)	not reported	240.0 ± 4.0g	240.58 ± 37.10 g	368.9 (1.3) g	184g	200g	284 g	150g	248 g	not reported	248 g	not reported	240.0 ± 4.0 g	229 g	not reported	252 g
Non carbon plated shoe stiffness	4.4 ± 1.8 N/m	19.1 ± 0.4N/mm	not reported	15.4 (1.0)	1.47 n/rad	not reported	not reported	not reported	7.0 Nm/rad	not reported	not reported	0.83 ± 0.02 n/mm	19.12 ± 0.43 n/mm	20.1 N/mm	0.65 N/mm and 0.76 N/mm	not reported
Carbon plated shoe model	experimental design with custom-made carbon fiber inserts	carbon fibre plate inserted in the middle of the midsole	Nike ZoomX Vaporlfy Next% 2	dding flat carbon fiber composite plates of 0.9 mm thickness under the shoe insoles	Nike ZoomX Vaporfly Next%	Nike ZoomX Vaporfly NEXT%	Nike ZoomX Vaporfly NEXT%	Nike Vaporfly 4%.	Nike Vaporfly	added a mid carbon plate beetween two layes of pebax	Nike Vaporfly Next% 2TM	carbon plate inserted below the insole	curved full carbon fiber plate inserted in the middle of the midsole	experimental shoes with curved carbon fiber plates added	exchanging the original insoles with custom-made carbon fiber insoles (3.2 mm)	Endorphin Speed 2 “super shoe”
Carbon plated shoe mass (g)	(added 28–35 g to the shoe)	248.3 ± 5.5g	201.08 ± 10.77 g	369.6 (2.1) g	186–200g	186g	188 g	213g ± 12	190g	not reported	176 g	not reported	248.3 ± 5.5 g	221 g	not reported	227 g,
Carbon plated shoe stiffness	four 1 mm thick carbon fiber inserts (6.4 ± 1.6 N/m)	35.2 ± 0.5N/mm	not reported	38.0 (1.8) n/mm	21.6n/mm	21.6n/mm	21.6n/mm	21.6n/mm	18.5 Nm/rad	not reported	not reported	2.63 ± 0.05 N/mm	35.21 ± 0.59 n/mm	43.1 N/mm	16.16 N/mm and 17.10 N/mm	not reported
Midsole material	not reported	foam	Pebax® foam	full polyurethane foam	PEBA (Pebax®)	polyether block amide midsole	ZoomX” foam made with new polyether block amide.	PEBA midsole foam	PEBA midsole foam	two layers of Pebax material	PEBA (Pebax®)	EVA MFOAM	ethylene-vinyl acetate [EVA]	foam	EVA	nylon plate and PWRRUN PB Peba
Speed (km/h)	8 km/h	3,000 m test - control: 18.28 km/h, increased LBS: 18.43 km/h	1,000 m - control: 17.82 km/h, iincreased LBS: 18.25 km/h	10.8 ± 1.1 km/h	16,63 km/h	15 km/h and 20 km/h	14 km/h	10 km/h - until exhaustion	16 km/h	13 km/h	12.9 km/h	12 km/h	9–17 km/h	13 km/h	12,6 km/h	9.4 km/h - 11.5 km/h
Type of evaluation	treadmill	outdoor athletics 400 m track	400 m outdoor track	outdoor incremental running test, an outdoor submaximal running test with physiological measurements, and an indoor submaximal running test with biomechanical measurements.	overground running on campus	treadmill	treadmill	treadmill	treadmill	treadmill	treadmill	treadmill	treadmill	treadmill	25 m long runway	treadmill
Type of study	randomized crossover study	randomized crossover study	randomized crossover study	randomized crossover study	randomized crossover study	randomized crossover study	randomized crossover study	randomized crossover study	randomized crossover study	randomized crossover study	randomized crossover study	crossover study	randomized crossover study	randomized crossover study	crossover study	randomized crossover study

Summary of the main characteristics of the randomized crossover studies included in this systematic review. The table details sample size, participant characteristics, footwear models, shoe mass and longitudinal stiffness, midsole composition, running speed and test environment, as well as biomechanical outcomes analyzed (e.g., joint kinematics, kinetics, and spatiotemporal parameters). All studies compared running shoes with embedded carbon-fiber plates against non-plated models, evaluating lower-limb mechanical variables such as joint moments and powers at the hip, knee, ankle, and metatarsophalangeal joints, leg stiffness, and ground reaction forces.

MTP, metatarsophalangeal; GRF, ground reaction force; ROM, range of motion.

### Risk of bias assessment

Methodological quality was evaluated using the *Downs and Black* checklist. Overall, the included studies demonstrated a low to moderate risk of bias and the average score was 15,83 (0-20 scale). All the studies were classified as low risk of bias. Most of the articles lacked reporting all the adverse events occurred with the subjects, the proportion the source population from which the subjects are derived, blinding those measuring the main outcomes, absence of sample size power calculations and there was no adjustment made in the final analysis when confounding was demonstrated. (Table 3).

**Table 3:** Methodological quality (downs & black index).

Study	Beltran et al	Catellanos-Salamanca et al. (1)	Catellanos-Salamanca et al. (2)	Flores et al	Hannigan et al	Hata et al. (1)	Hata et al. (2)	Hebert-Losier et al	Hoogkamer et al	Liu et al	Martinez et al	Paradisis et al.	Perrin et al	Rodrigo-Carranza et al. (1)	Rodrigo-Carranza et al. (2)	Willwatcheret al
1	1	1	1	1	1	1	1	1	1	1	1	1	1	1	1	1
2	1	1	1	1	1	1	1	1	1	1	1	1	1	1	1	1
3	1	1	1	1	1	1	1	1	1	1	1	1	1	1	1	1
4	1	1	1	1	1	1	1	1	1	1	1	1	1	1	1	1
5	1	1	1	1	0	1	1	1	1	1	1	1	1	1	1	1
6	1	1	1	1	1	1	1	1	1	1	1	1	1	1	1	1
7	1	1	1	1	1	1	1	1	1	1	1	1	1	1	1	1
8	0	0	1	0	0	0	0	0	0	0	0	0	0	0	0	0
9	1	1	1	1	1	1	1	1	1	1	1	1	1	1	1	1
10	0	0	0	0	0	0	0	0	0	0	0	0	0	0	0	0
11	1	1	1	1	1	1	1	1	1	1	1	1	1	1	1	1
12	0	0	0	0	0	0	0	0	0	0	0	0	0	0	0	0
13	1	1	1	1	1	1	1	1	1	1	1	1	1	1	1	1
14	1	1	1	1	1	1	1	1	1	1	1	1	1	1	1	1
15	1	1	1	1	1	1	1	1	1	1	1	1	1	1	1	1
16	1	1	1	1	1	1	1	1	1	1	1	1	1	1	1	1
17	1	1	1	1	1	1	1	1	1	1	1	1	1	1	1	1
18	1	1	1	1	1	1	1	1	1	1	1	1	1	1	1	1
19	0	0	0	0	1	1	1	1	1	1	1	1	1	1	1	0
20	0	0	0	0	0	0	0	0	0	0	0	0	0	0	0	0
Total	15	15	16	15	15	16	16	16	16	16	16	16	16	16	16	15

Item-level scores (0–1) across 20 items grouped as reporting (1–9), external validity (10–11), internal validity (12–15), and selection bias (16–20). Total score 0–20; risk-of-bias categories: 0–6 high, 7–13 moderate, 14–20 low. The mean total score across studies was 15.73; All 15 studies were classified as low risk of bias.

### Certainty of evidence

Certainty of evidence was assessed using the *GRADE* approach. For the analyzed outcomes, most studies showed a low risk of bias and no relevant issues regarding indirectness. However, some limitations were identified concerning inconsistency of results, particularly for leg stiffness, positive knee power, and metatarsophalangeal (MTP) joint power, due to heterogeneity across studies. Consequently, the certainty of evidence was rated as moderate for stride frequency and hip/ankle power, but low for leg stiffness, knee power, and MTP power. These findings suggest that results should be interpreted with caution, although the available studies were methodologically sound. (Table 4).

**Table 4:** GRADE assessment of certainty of evidence for each primary outcome.

Outcome	Step frequency (steps/min)	Leg stiffness (kN/m)	Peak positive ankle power (W/kg)	Peak positive knee power (W/kg)	Peak positive hip power (W/kg)	Peak positive MTP power (W/kg)
N° of Reviews (Studies)	8 Randomized crossover studies and 1 crossover study	5 Randomized crossover studies and 1 crossover study	5 Randomized crossover studies	4 Randomized crossover studies	4 Randomized crossover studies	3 Randomized crossover studies
Study Design	Biomechanical comparative studies	Biomechanical comparative studies	Biomechanical comparative studies	Biomechanical comparative studies	Biomechanical comparative studies	Biomechanical comparative studies
Risk of Bias	Not serious	Not serious	Not serious	Not serious	Not serious	Not serious
Inconsistency	Serious (moderate heterogeneity)	Serious (heterogeneous effects)	Not serious	Serious (inconsistent estimates)	Not serious	Serious (heterogeneous effects)
Indirectness	Not serious	Not serious	Not serious	Not serious	Not serious	Not serious
Imprecision	Serious (CI includes null)	Serious (imprecise CI)	Serious (wide CI)	Serious (wide CI)	Serious (borderline CI)	Serious (imprecise estimates)
Publication Bias	Undetected	Undetected	Undetected	Undetected	Undetected	Undetected
Overall Certainty	⬤⬤◯◯ Low	⬤⬤◯◯ Low	⬤⬤⬤◯ Moderate	⬤⬤◯◯ Low	⬤⬤⬤◯ Moderate	⬤⬤◯◯ Low

Judgments are based on risk of bias, inconsistency, indirectness, imprecision, and publication bias. Overall certainty was rated as high (⬤ ⬤ ⬤ ⬤), moderate (⬤ ⬤ ⬤ ◯), low 277 (⬤ ⬤◯◯), or very low (⬤◯◯◯) according to GRADE criteria.

### Meta analysis

As previously discussed, the Standardized Mean Difference (SMD) with 95% confidence intervals (CIs) was calculated for each outcome. When multiple bending stiffness in shoes with carbon plates were used, we extracted the stiffest of them all to use as the comparator.

### Leg stiffness (kN/m)

**Figure 2:**
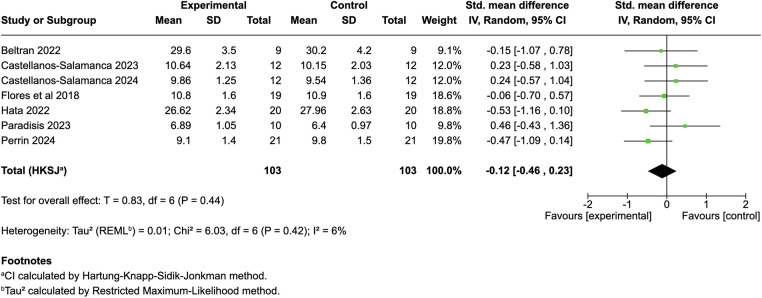
Leg stiffness (kN·m^−1^) in carbon-plated vs. non-carbon-plated running shoes. Forest plot showing the standardized mean difference (SMD) in leg stiffness between running shoes equipped with carbon-fiber plates (CPS) and non-plated shoes (NCPS). Random-effects model with Hartung– Knapp–Sidik–Jonkman (HKSJ) adjustment was applied. Heterogeneity was low (I^2^ = 0%).

The pooled analysis (1, 2, 17, 23, 33, 34, 36) showed no significant effect of carbon-plated shoes on leg stiffness (SMD = −0.12; 95% CI: −0.46 to 0.23; *p* = 0.44). Heterogeneity was low (I² = 6%), indicating consistent findings across studies Figure 2.

### Peak positive ankle power (W/kg)

**Figure 3:**
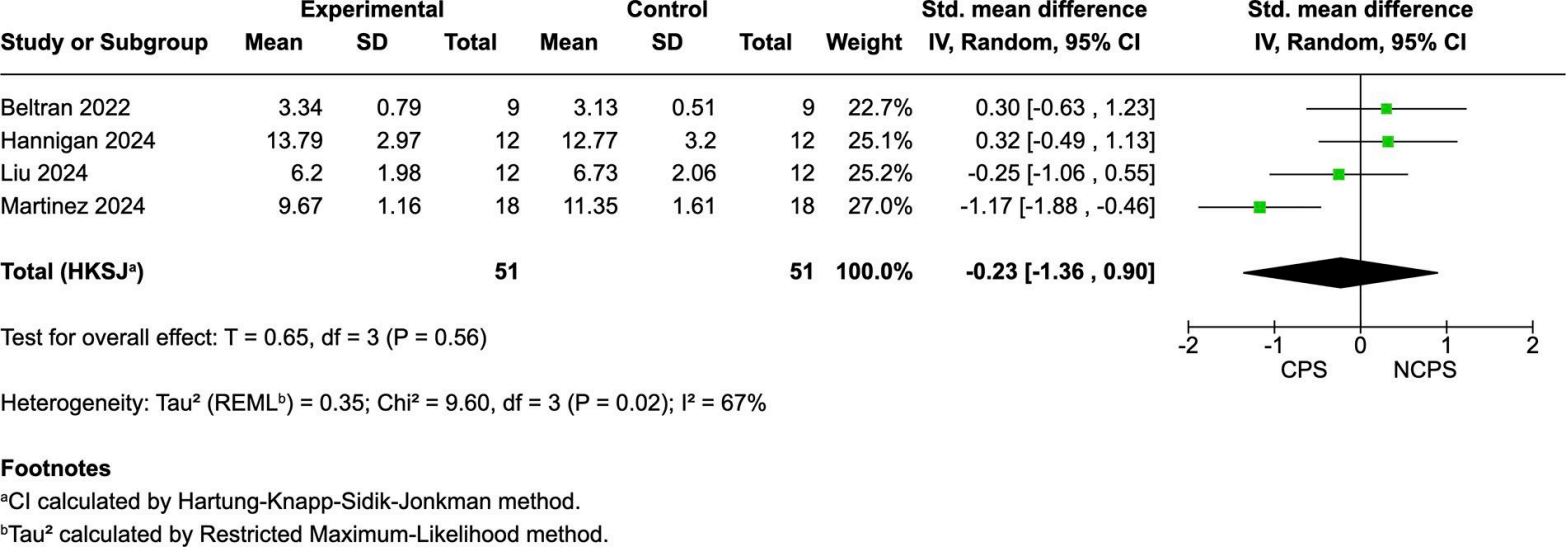
Peak positive ankle power (W·kg^−1^) in carbon-plated vs. non-carbon-plated running shoes. Forest plot comparing peak positive ankle power between CPS and NCPS conditions. Results are expressed as standardized mean differences (SMD) with 95% confidence intervals, calculated under a random- effects model (HKSJ).

The pooled analysis of five studies (3, 11, 36–38) (*n* = 122) demonstrated a borderline statistically significant difference between groups in ankle power, favoring the CPS group (SMD = −0.71; 95% CI: −1.42 to 0.00; *p* = 0.05). Moderate heterogeneity was observed across studies (I² = 43%) Figure 3.

### Peak positive hip power (W/kg)

**Figure 4:**
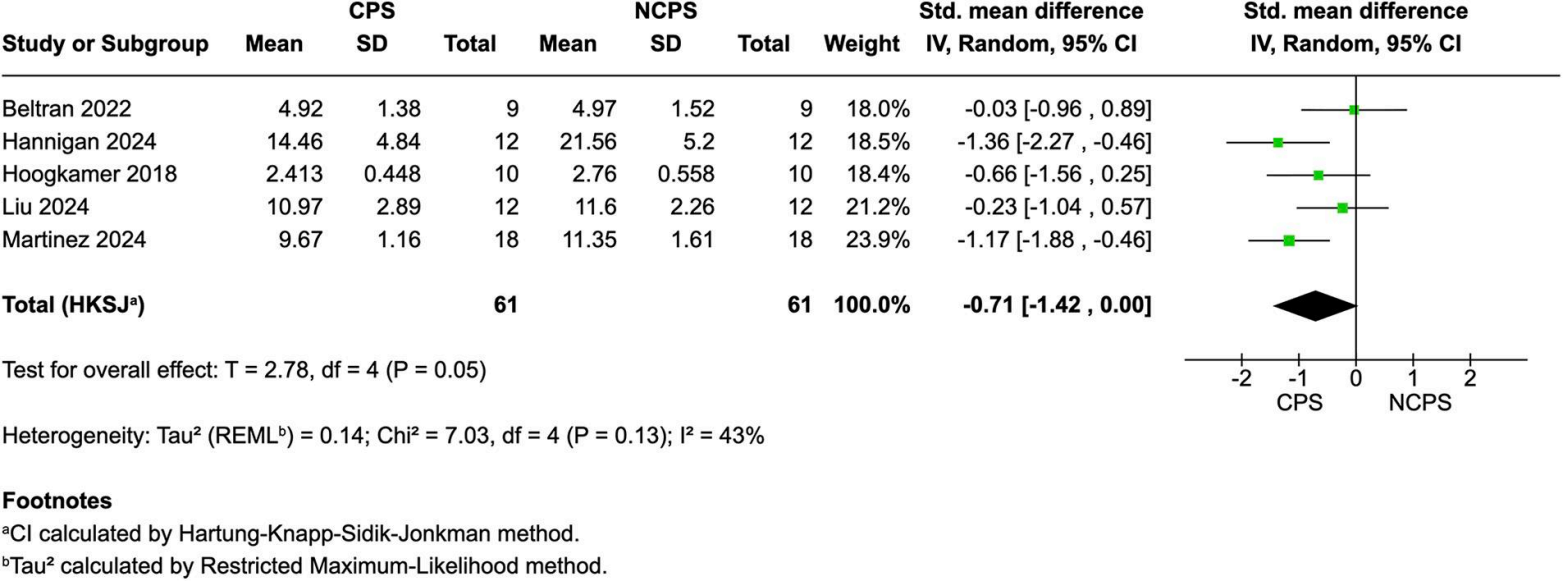
Peak positive hip power (W·kg^−1^) in carbon-plated vs. non-carbon-plated running shoes. Forest plot showing pooled differences in peak positive knee power between CPS and NCPS conditions. Estimates were derived using a random-effects model with HKSJ correction. Heterogeneity was negligible (I^2^ = 0%), suggesting consistent results across studies.

The pooled analysis of four studies (11, 36–38) (*n* = 102) showed no statistically significant difference between groups for knee power (SMD = 0.21; 95% CI: −0.10 to 0.52; *p* = 0.12). No heterogeneity was observed among the included studies (I² = 0%) Figure 4.

### Peak positive knee power (W/kg)

**Figure 5:**
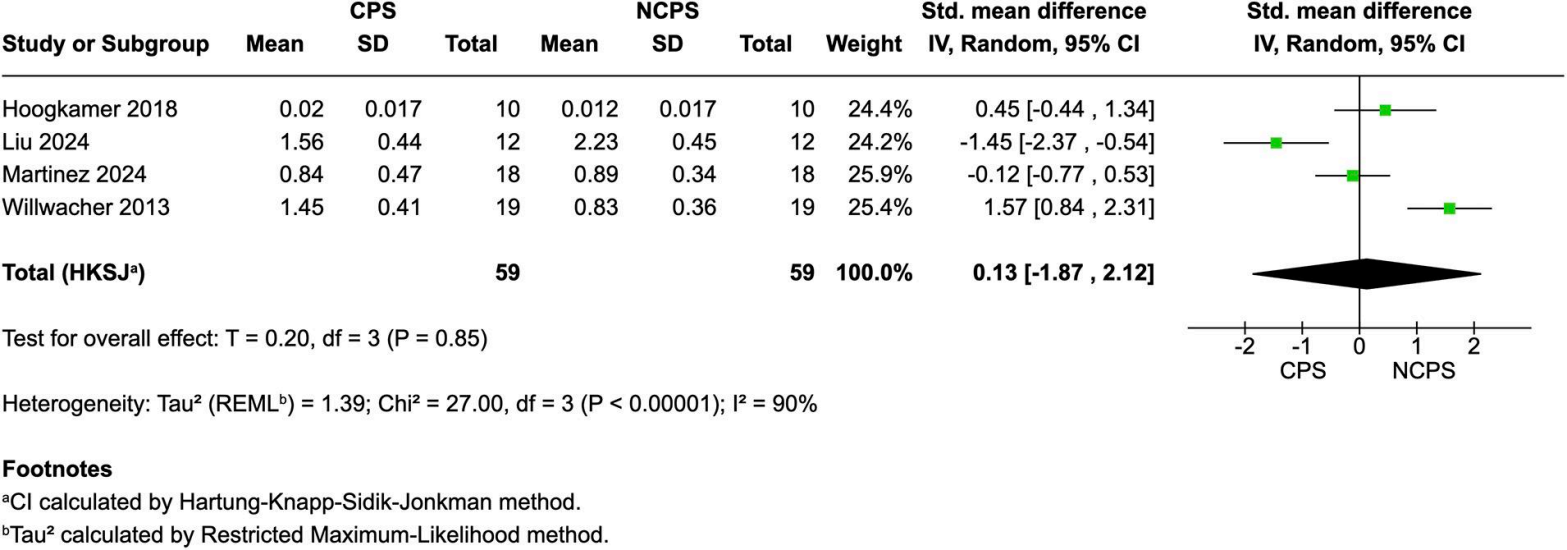
Peak positive knee power (W·kg^−1^) in carbon-plated vs. non-carbon-plated running shoes. Meta-analysis comparing peak positive hip power between CPS and NCPS. Random-effects model (HKSJ) with Restricted Maximum Likelihood (REML) variance estimation was used. Confidence intervals indicate no significant between-condition difference (*p* > 0.05).

The pooled analysis of four studies (11, 36–38) (*n* = 102) showed no statistically significant difference between groups for hip power (SMD = −0.23; 95% CI: −1.36 to 0.90; *p* = 0.56). Substantial heterogeneity was observed among the included studies (I² = 67%) Figure 5.

### Peak positive metatarsophalangeal (MTP) power (W/kg)

**Figure 6:**
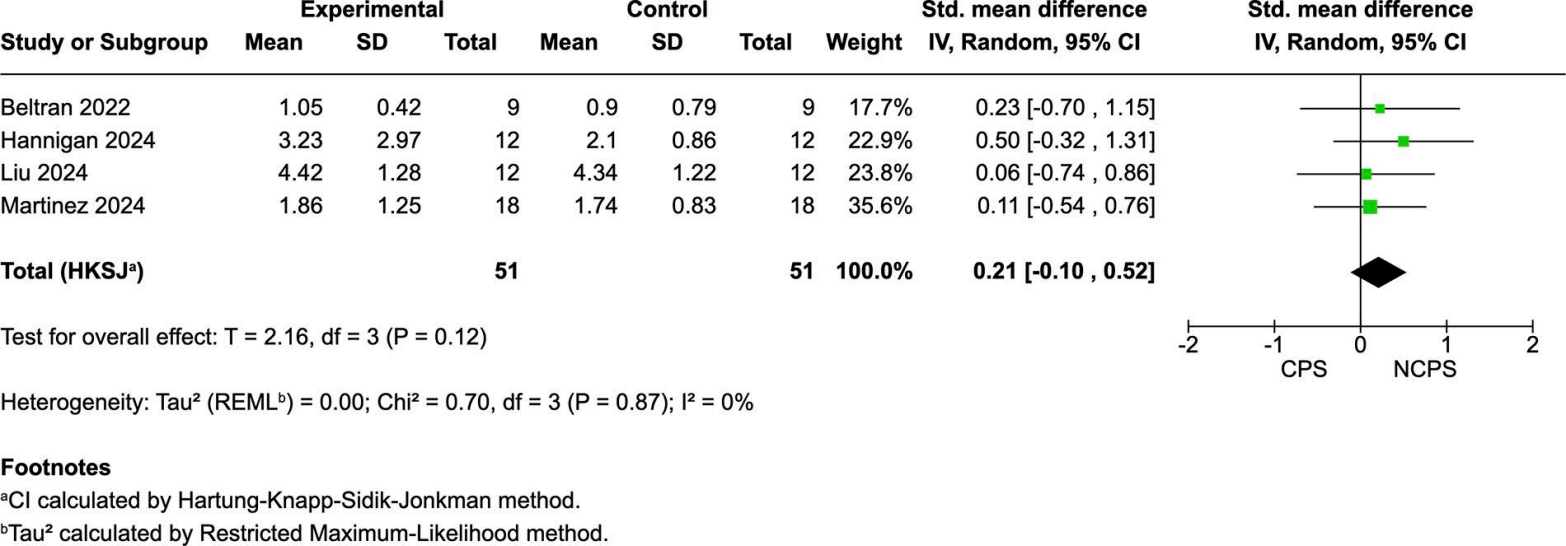
Peak positive metatarsophalangeal (MTP) power (W·kg^−1^) in carbon-plated vs. non-carbon-plated running shoes. Forest plot comparing peak positive MTP joint power between CPS and NCPS. The pooled standardized mean difference (SMD) was estimated via a random-effects model (HKSJ).

The pooled analysis of four studies (3, 11, 38, 39) (*n* = 118) showed no statistically significant difference between groups for MTP power (SMD = 0.13; 95% CI: −1.87 to 2.12; *p* = 0.85). Considerable heterogeneity was observed among the included studies (I² = 90%) Figure 6.

### Stride frequency (steps/min)

**Figure 7:**
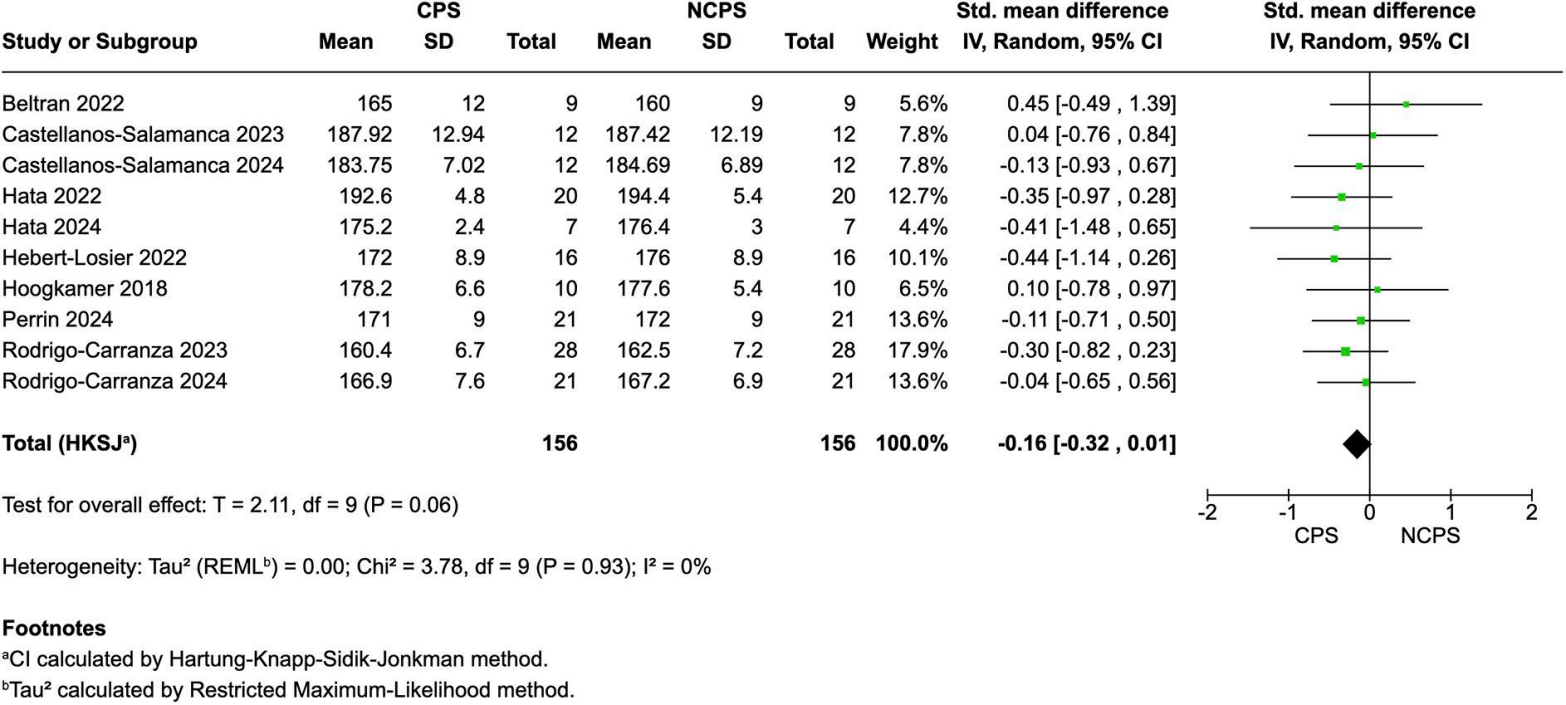
Step frequency (steps·min^−^¹) in carbon-plated vs. non-carbon-plated running shoes. Forest plot summarizing step frequency differences between CPS and NCPS. Pooled effect sizes standardized mean difference (SMD ± 95% CI) were calculated with a random-effects HKSJ model.

The pooled analysis of ten studies (1, 3, 16, 17, 22, 23, 26, 29, 34, 36) (*n* = 312) showed no statistically significant difference between groups for step frequency (SMD = −0.16; 95% CI: −0.32 to 0.01; *p* = 0.06). No heterogeneity was observed among the included studies (I² = 0%) Figure 7.

## Discussion

In this systematic review and meta-analysis, we evaluated the effects of carbon-plated CPS on running biomechanics compared with NCPS, focusing on joint positive power (hip, knee, ankle, and MTP), leg stiffness, and stride frequency. Overall, the findings indicate that CPS do not meaningfully modify lower-limb joint power or leg stiffness, while a small but significant reduction in stride frequency was observed.

### Ankle power

In accordance with the hypothesis that increased longitudinal bending stiffness would reduce ankle mechanical output through restricted dorsiflexion and altered lever mechanics (10), our pooled results showed significant differences between CPS and NCPS. In Martinez et al. (11) study, reduced energy generation was found, which may indicate decreased energetic demand of the plantarflexor muscles, potentially contributing to a lower metabolic cost of contraction (9). Similarly, diminished positive ankle work has been reported in male runners (3) and may be attributed to reductions in plantarflexion angular velocity, ankle plantarflexor torque, or a combination of both factors.

### Hip power

A non-significant trend toward higher hip power was observed in CPS conditions. Although some studies report that proximal joints may assume a compensatory role when footwear stiffness increases, particularly under fatigue (2), the current evidence across controlled laboratory conditions does not support systematic redistribution toward the hip. Differences in running speed, adaptation time, and individual technique may partly explain the variability across studies.

### Knee power

Knee power did not differ between footwear conditions, consistent with prior research showing that modifications in bending stiffness primarily influence distal structures, namely the ankle and MTP joints, while sparing the knee (12, 13). Studies on enhanced longitudinal stiffness report modest shifts in mechanical work distribution but generally describe the knee as biomechanically stable across conditions (5, 14).

### MTP joint power

Although the MTP joint is theoretically the most directly affected by carbon-plate stiffness, our analysis found no significant differences and substantial heterogeneity. Differences in plate geometry, curvature, thickness, and midsole design across footwear models (15, 24, 28) likely contribute to inconsistent findings, compounded by the limited number of studies evaluating this parameter.

### Leg stiffness

Leg stiffness was not significantly different between CPS and NCPS, indicating that increased shoe stiffness does not translate into systemic changes in the leg-spring mechanism. Prior research similarly reports stable leg stiffness across footwear conditions (16, 17), suggesting that runners maintain their preferred stiffness strategy through neuromuscular adjustments rather than modifying global lower-limb compliance (15, 18). This stability reinforces the idea that CPS effects are more likely to appear in temporal or kinematic variables than in mechanical stiffness metrics.

### Stride frequency

The negative effect presented on the meta-analysis suggests that, on average, the CPS group presents a slightly lower step frequency compared to the NCPS group. However, this difference is very small and statistically uncertain. This finding aligns with evidence that increased longitudinal stiffness and compliant foams may enable runners to maintain speed with slightly longer strides (3, 13, 15). Although some studies report no meaningful change in cadence (16, 19), as shown in our results, differences across studies may reflect variations in running intensity, population characteristics, or shoe construction. Reduced cadence at constant speed implies longer stride length, which may represent a strategy to capitalize on increased energy return rather than a detrimental change in gait. Therefore, the insignificant statistics results imply that the magnitude of cadence change was small, reinforcing that CPS effects are subtle rather than transformative at the biomechanical level.

### Integrated interpretation

Within the biomechanical scope of the present analysis, carbon-plated running shoes did not systematically alter positive joint power or leg stiffness when compared with non-carbon-plated shoes. Across the heterogeneous body of evidence, the most consistently observed adaptation was the reduction of ankle power in carbon plated shoes, whereas remaining joint-level mechanics and spatiotemporal mechanical changes were not detected. These results suggest that, within the biomechanical variables analyzed, carbon-plated footwear may influence running primarily through increasing the efficiency of ankle plantarflexion movement, as it was evidenced that the concentric MEC improved without any increase in the MEE and/or joint torque (5). However, factors such as plate-foam interaction, individual running strategy, and running speed likely modulate these responses, contributing to the variability and inconsistencies reported across studies (9, 10, 15, 18, 20).

### Methodological heterogeneity and biomechanical comparability of outcomes

An important methodological consideration when interpreting the present meta-analysis is the biomechanical comparability of pooled outcomes across studies. Although joint power and leg stiffness are commonly reported variables in running biomechanics, their absolute values and functional interpretation can vary substantially depending on modelling assumptions. Differences in inverse dynamics approaches, marker sets and segment definitions, foot models (single- vs. multi-segment), signal filtering strategies, testing conditions (treadmill vs. overground), and definitions or normalization of joint power may all influence outcome estimates.

To mitigate this variability, the present meta-analysis focused on within-subject comparisons between carbon-plated and non-carbon-plated footwear, thereby reducing the influence of inter-individual anthropometric and neuromuscular differences. Nevertheless, even relative differences may remain sensitive to modelling choices, particularly for distal joints such as the metatarsophalangeal (MTP) joint. MTP power is especially dependent on foot model complexity, joint coordinate system definition, and marker placement, which likely contributes to the substantial heterogeneity observed for this outcome.

As a result, the pooled estimates should be interpreted as reflecting general trends rather than precise biomechanical equivalence across studies. The absence of consistent effects on most of the joint powers or leg stiffness therefore suggests that carbon-plated footwear does not induce robust, directionally consistent changes that are detectable across diverse biomechanical modelling frameworks. However, smaller or model-specific effects cannot be excluded and may be masked by methodological heterogeneity.

An additional source of heterogeneity relates to how biomechanical variables are quantified and reported across studies. Some investigations assess leg stiffness using normalized or dimensionless outputs derived from spring-mass models, rather than reporting absolute values (kN·m^−^¹). Similarly, spatiotemporal variables may be expressed as relative changes or interaction effects without providing extractable means and measures of variability. While such approaches are biomechanically valid within individual studies, they limit comparability and preclude quantitative synthesis within a meta-analytic framework. Consequently, studies reporting biomechanical outcomes exclusively in these formats could not be included in the present meta-analysis, despite their conceptual relevance.

Future studies would benefit from greater standardization of biomechanical modelling approaches or from coordinated multi-laboratory efforts using harmonized protocols. Such strategies would improve the interpretability of pooled biomechanical outcomes and facilitate clearer links between footwear design, joint-level mechanics, and functional or energetic adaptations.

### Biomechanics vs. energetics: interpretation of findings

An important consideration in interpreting these findings is the distinction between biomechanical and energetic adaptations to carbon-plated footwear. Although carbon-plated shoes have consistently been associated with improvements in running economy and reduced metabolic cost (7), the present meta-analysis did not identify systematic changes in joint power or leg stiffness. The only consistent biomechanical adaptation observed was a small reduction in step frequency.

This apparent dissociation suggests that metabolic benefits may arise from mechanisms not fully captured by peak joint power or global stiffness measures. Improvements in muscle-tendon efficiency, altered fascicle behavior, or enhanced energy return at the shoe-ground interface may reduce energetic cost without requiring substantial redistribution of joint-level mechanics.

Therefore, the absence of consistent biomechanical differences should not be interpreted as a lack of performance relevance. Instead, it indicates that carbon-plated footwear may enhance running efficiency through integrated neuromuscular and material-level mechanisms while preserving overall joint mechanics.

### Clinical implications

Understanding how carbon-plated footwear influences ankle power and other biomechanical variables has relevant clinical and practical implications, particularly in the context of running performance, injury prevention, and rehabilitation. In this review, it was the only variable that showed a significant change when runners used carbon-plated shoes, whereas the remaining joint powers, stride frequency and leg stiffness remained largely unaltered. This pattern suggests that the biomechanical effects of carbon-plated shoes are likely joint-specific rather than global. Additionally, the primary mechanical adaptation occurs at the distal joint level, indicating that carbon-plated shoes alter propulsion mechanics by redistributing work away from the ankle without substantially modifying overall running rhythm or global limb stiffness.

A lower ankle power could possibly decrease positive work performed by the triceps sural, as it was evidenced in previous study (12) that the gastrocnemius medialis muscle energy cost was lower with stiffer conditions of midsole bending stiffness. Accordingly, Cigoja et al. (12) concluded that running in stiffer shoes, like carbon plated shoes, the plantarflexor muscle–tendon unit (primarily the gastrocnemius and soleus muscles in conjunction with the Achilles tendon) operates under more mechanically favorable conditions. As a result, those mechanical implications could be advantageous in rehabilitation settings such as Achilles tendinopathy or chronic calf overload. However, direct clinical evidence supporting this protective effect remains limited.

A reduction in step frequency is associated with longer stride length and potentially longer ground contact time at a constant running speed (18). These changes can modify how impact forces are distributed across the lower limb. For some runners, especially those with a predisposition to overstriding or reduced neuromuscular control, a lower cadence may increase loading at the tibia, patellofemoral joint, or hip (7, 11, 21, 31). Clinicians should therefore consider whether cadence reductions induced by CPS might exacerbate symptoms in individuals with stress-related tibial injuries, patellofemoral pain, or hip tendinopathies.

On the other hand, the energy return characteristics associated with CPS and compliant midsoles may allow certain athletes, particularly those with efficient running mechanics, to maintain speed with reduced mechanical cost (10, 15). For these runners, a slightly longer stride may not elevate injury risk and may even improve perceived exertion or running economy. This underscores the need for individualized assessment rather than universal recommendations regarding CPS use.

The absence of changes in leg stiffness and knee, hip and MTP power may also have practical implications. Since these variables remained stable across shoe conditions, CPS do not appear to systematically alter most of lower-limb spring behavior or joint-level mechanical output. This stability may reduce the likelihood of abrupt biomechanical changes that could predispose runners to injury. However, runners transitioning to CPS should still be monitored during early adaptation phases, as small deviations in cadence or foot placement can accumulate over longer training cycles.

Finally, because CPS are increasingly used in both training and competition, clinicians should be aware that their effects may differ between recreational and highly trained runners. Performance level, experience with CPS, and running speed likely influence spatiotemporal adaptations. Tailoring footwear recommendations to runner profile, training goals, injury history, and biomechanical characteristics may therefore improve safety and optimize long-term performance outcomes.

### Limitations

Despite including 16 studies, the number of meta-analyzable outcomes was smaller due to methodological heterogeneity. Variations in plate stiffness, midsole foams, shoe mass, running speeds, and participant characteristics limited the ability to isolate the specific effects of carbon plates. Sample sizes were generally small and predominantly male, reducing generalizability. Moreover, factors such as foot strike pattern and prior CPS experience were not consistently reported, despite their potential influence on biomechanical responses.

Future research should prioritize standardized protocols, larger and more diverse samples, and longitudinal designs assessing adaptation over time. Comprehensive biomechanical analyses, including joint moments, 3D kinematics, muscle activity, and plantar pressure, would help clarify the mechanisms underlying CPS-induced gait modifications. Stratifying analyses by runner characteristics (e.g., performance level, habitual cadence) may also reveal subgroups that benefit most, or least, from CPS.

## Conclusion

This systematic review and meta-analysis indicate that carbon-plated running shoes do not produce consistent changes in hip, knee, MTP power, leg stiffness or step frequency when compared with non-carbon-plated shoes, while being associated with a reduction in ankle power. Within the variables analyzed, these findings suggest that biomechanical adaptations to carbon-plated footwear may be primarily localized at the distal joint level rather than reflecting widespread alterations across the lower-limb kinetic chain. The absence of systematic changes in proximal joint mechanics and global spatiotemporal parameters further indicates that the mechanical effects of carbon-plated shoes are not uniformly distributed across joints. Importantly, because energetic and physiological variables were not included, the present results cannot elucidate the mechanisms underlying the well-documented metabolic advantages of carbon-plated footwear, highlighting the need for future integrative studies combining biomechanical and energetic outcomes.
